# Community health workers at the dawn of a new era: 6. Recruitment, training, and continuing education

**DOI:** 10.1186/s12961-021-00757-3

**Published:** 2021-10-12

**Authors:** Meike J. Schleiff, Iain Aitken, Mohammad Ariful Alam, Zufan Abera Damtew, Henry B. Perry

**Affiliations:** 1 Health Systems Program, Department of International Health, Johns Hopkins Bloomberg School of Public Health, Baltimore, MD USA; 2 Management Sciences for Health, Ministry of Public Health, Kabul, Afghanistan; 3 BRAC, Dhaka, Bangladesh; 4 EngenderHealth, Addis Ababa, Ethiopia

**Keywords:** Community health worker, Training, Education, Continuing education, Health workforce

## Abstract

**Background:**

This is the sixth of our 11-paper supplement entitled “Community Health Workers at the Dawn of New Era”. Expectations of community health workers (CHWs) have expanded in recent years to encompass a wider array of services to numerous subpopulations, engage communities to collaborate with and to assist health systems in responding to complex and sometimes intensive threats. In this paper, we explore a set of key considerations for training of CHWs in response to their enhanced and changing roles and provide actionable recommendations based on current evidence and case examples for health systems leaders and other stakeholders to utilize.

**Methods:**

We carried out a focused review of relevant literature. This review included particular attention to a 2014 book chapter on training of CHWs for large-scale programmes, a systematic review of reviews about CHWs, the 2018 WHO guideline for CHWs, and a 2020 compendium of 29 national CHW programmes. We summarized the findings of this latter work as they pertain to training. We incorporated the approach to training used by two exemplary national CHW programmes: for health extension workers in Ethiopia and *shasthya shebikas* in Bangladesh. Finally, we incorporated the extensive personal experiences of all the authors regarding issues in the training of CHWs.

**Results:**

The paper explores three key themes: (1) professionalism, (2) quality and performance, and (3) scaling up. *Professionalism:* CHW tasks are expanding. As more CHWs become professionalized and highly skilled, there will still be a need for neighbourhood-level voluntary CHWs with a limited scope of work. *Quality and performance:* Training approaches covering relevant content and engaging CHWs with other related cadres are key to setting CHWs up to be well prepared. Strategies that have been recently integrated into training include technological tools and provision of additional knowledge; other strategies emphasize the ongoing value of long-standing approaches such as regular home visitation. *Scale-up:* Scaling up entails reaching more people and/or adding more complexity and quality to a programme serving a defined population. When CHW programmes expand, many aspects of health systems and the roles of other cadres of workers will need to adapt, due to task shifting and task sharing by CHWs.

**Conclusion:**

Going forward, if CHW programmes are to reach their full potential, ongoing, up-to-date, professionalized training for CHWs that is integrated with training of other cadres and that is responsive to continued changes and emerging needs will be essential. Professionalized training will require ongoing monitoring and evaluation of the quality of training, continual updating of pre-service training, and ongoing in-service training—not only for the CHWs themselves but also for those with whom CHWs work, including communities, CHW supervisors, and other cadres of health professionals. Strong leadership, adequate funding, and attention to the needs of each cadre of CHWs can make this possible.

Key messages: Summary **Key findings**

1. Training is a comprehensive and dynamic element of CHW programmes that needs to be well funded and professionalized.
2. Training must be seen as much more than just pre-service training, but rather as ongoing iterative training.
3. Training for effective CHW programmes also needs to be seen not only as training of CHWs, but also training the community, supervisors of CHWs, and others within the health system in order to help these stakeholders appreciate and understand and make effective use of CHWs.

**Key implications**

1. Professionalism of CHW training means ongoing assessment of quality and continuous quality improvement, which will require programmes to have adequate capacity and resources for these activities.
2. CHW roles will continue to change over time, and therefore ongoing and dynamic training updates will be an essential element of an effective CHW programme. This includes adding new evidence-based interventions and approaches in response to population health needs. Public health emergencies, such as the COVID-19 pandemic, bring further and urgent attention to the need for ongoing and responsive training of CHWs.
3. CHWs are increasingly recognized as key contributors and components of strong primary health care systems; training is a key prerequisite and incentive for CHWs to become and remain effective. Therefore, ensuring that training for CHWs is part of the larger health workforce strategy and priorities is essential for achieving the Sustainable Development Goals and universal healthcare.
4. A strategy for the growth and development of CHWs is needed, so that they have an opportunity to develop themselves through a clear career structure.

## Background

The roles of CHWs have changed substantially over recent decades as the services they provide and the specific groups that they have commonly focused on—historically, mostly children and women of childbearing age—have also expanded. CHW responsibilities now may also include newborns, subpopulations with other infectious diseases, noncommunicable diseases (NCDs), and mental health as well as other services such as registration of vital events, disease surveillance, and response to humanitarian disasters and pandemics (including COVID-19) [1–4]. CHW programmes have seen a gradual shift from comprising mostly lay workers providing health promotion and education and linking communities to services offered by other health professionals, to becoming first-line providers for many services. These may include provision of immunizations, injectable contraceptives, vitamin A supplementation, and deworming medicine; diagnosis and treatment of childhood pneumonia, diarrhoea, and undernutrition using protocols for integrated community case management (iCCM); the provision of commodities including contraceptives and insecticide-treated bed nets; the detection and treatment of tuberculosis (TB) patients; and working in a range of vertical programmes such as those to control HIV and malaria [5–7]. Many CHW programmes have also improved maternal and child health through their support for antenatal care, safe delivery, postnatal care, and home-based care of the newborn [8–10]. The roles that CHWs are currently providing in large-scale CHW programmes is discussed further in Paper 5 in this series on roles and tasks [11].

In recent years, health systems are increasingly facing epidemiological and demographic transitions and also crises such as climate change, conflict, and outbreaks like the current COVID-19 pandemic. This is pressuring health system leaders to look for strategies to respond to these complex and often long-term care needs of their populations [12–14]. While much of the health systems strengthening literature does not explicitly identify community or CHW roles [15], CHWs are providing an increasing range of care, managing more complex health issues, collaborating more closely with other health workers, and helping to better link communities with health systems as part of a better integrated health system [16, 17].

All of these shifts have direct influences on the selection criteria and the duration, content, and modalities of pre-service and in-service training for CHWs. Training has been identified as an important factor for CHW programme successes as well as a barrier when not adequately provided. While very important, the broader literature on training of health workers in low- and middle-income countries [18, 19] suggests that training CHWs in isolation from the context in which they work is not likely to improve their performance, and even if combined with supervision and group problem-solving approaches, their effects on provider quality of care may not be as significant as might be expected.

The recent WHO guideline on health policy and system support for CHW programmes break out guidance on selection criteria, duration of pre-service training, competencies for curriculum, modalities of training, and certification [20]. While all of the recommendations are conditional on the specific programme context and objectives, it is clear from the guideline that decisions related to the kind and extent of CHW training must reflect the kinds of roles and tasks they will perform, which in many contexts, are becoming more extensive and more highly integrated into the health system [21].

Recognizing the recent renewed interest in CHW programmes in light of the Sustainable Development Goals (SDGs) and the goal of achieving Universal Health Coverage (UHC) by 2030, there is intensified attention on how to optimize and scale these programmes [22, 23]. This paper aims to provide actionable guidance for practitioners and researchers, based on the current literature and our experiences, regarding training related to CHW programmes in the years ahead. This is the sixth paper of an 11-paper series [11, 24–32] concerning the growing importance of CHW programmes and their potential significance for contributing to an acceleration of progress in achieving global health goals.

This paper builds on Chapter 9, “Training Community Health Workers for Large-Scale Community-Based Health Care Programs”, written by one of us (IA) in the 2014 publication entitled *Developing and Strengthening Community Health Worker Programs at Scale: A Reference Guide for Program Managers and Policy Makers* [33] (which we refer to as the CHW Reference Guide). That chapter synthesized extensive evidence to date and laid out several takeaway messages: (1) tailor the training to the context as well as to the ongoing needs of individual CHWs rather than design “one-size-fits-all” trainings; (2) learn from and build on what is working, (3) draw from examples of diverse exemplary cases of how training has been accomplished; and (4) ensure a comprehensive training package that integrates pre-service training with in-service training of CHWs, training of supervisors of CHWs, training of communities for their roles, and training of others in the health system about CHWs (the roles and responsibilities of CHWs) and their value to the health system.

Here, we update the contents of that 2014 chapter to provide a summary of the current status of training CHWs in order to understand the new status quo. Also, we identify a set of current priorities for additional actions and research related to training CHWs—and other workers involved in supporting primary healthcare (PHC) and community health programmes such as supervisors, clinicians, and managers—based on current evidence regarding the approaches to training now being utilized by national CHW programmes throughout the world [34]. Finally, we consider training from a broader perspective as well—not only the training needs of CHWs but the training needs for all those involved with the CHW programme—in order to sustainably enhance the quality and performance of the entire programme. We utilize case examples and current literature to explore the opportunities and challenges that each priority issue presents.

## Methods

As mentioned above, we updated and expanded a chapter from a previous book on training of CHWs in large-scale programmes [35]. We explored the limited existing peer-reviewed literature as well as grey literature pertaining the recruitment and training of CHWs. We also relied on the extensive personal experience of the authors related to training of CHWs along with their wide knowledge of this topic as a result of long-standing work in this area. We reviewed the systematic review of reviews concerning CHWs to ascertain publications related to the training of CHWs [4, 36]. We reviewed the 2018 WHO Guideline for CHW programmes to include their recommendations regarding training [37]. Most importantly, we relied on recently published information from a recently published compendium of 29 national CHW programmes entitled *Health for the People: National Community Health Programs from Afghanistan to Zimbabwe* [21]. All of the case studies followed the same format in terms of topics addressed, and one of those topics is “selection and training”. To our knowledge, this is the most detailed current information about national CHW programmes that is available at present.

## Results

### Current status of training in national CHW programmes around the world

A recently published compendium of 29 case studies (including one example of a failed national CHW programme) provides an array of experiences regarding training CHWs themselves (Table 1). The duration, modalities, and main content varies widely, from just a few days or weeks up to as much as 3 years (for Nigeria’s community health extension workers [CHEWs]). In Paper 1 of this series [24], we classify CHWs into three categories: community health volunteers (CHVs), health extension workers (HEWs), and auxiliary health workers. CHVs have only a few days of training and may receive some intermittent per diem payments or other forms of limited remuneration and/or other informal incentives such as recognition and respect from their community. Higher-level CHWs are more extensively trained and may receive a regular salary. We have delineated our discussion of priority issues and opportunities by these categories of CHWs where appropriate.

**Table 1:** Recruitment and training for CHWs from 29 countries [21]

Country	Name of CHW cadre	Selection criteria and process	Role and tasks performed	Duration and nature of training
Afghanistan	CHW	Selected through a consultation process between the NGO staff and the community elders 18+ years of age (no upper age limit) Respected by other community members No educational requirement	Health promotion, iCCM, family planning counselling, provision of contraceptives, screen and treat TB (DOTS), first aid, maintain map of households in catchment area, report vital events, and send report to the national HIS	17 weeks (three separate 3-week classroom modules and two separate 1-month field experiences)
Bangladesh	*Shasthya shebika* (BRAC [Building Resources Across Communities])	Identification of prospective service supervisors is made by the local Village Health and Development Committee; final selection is made by BRAC staff together with local village leaders and government officials Women only Age 25–40 Married with no children younger than 5 years Motivated Have at least 8 years of schooling Does not live near a healthcare facility or a large bazaar	Health promotion, pregnancy identification, family planning, treatment of uncomplicated acute illnesses, referral for immunization, screen and treat TB (DOTS), screen for presbyopia and sell glasses, community mobilization	3 weeks of basic classroom training followed by refresher trainings lasting 1–3 days every few months
	*Shasthya kormi* (BRAC)	Preferably be a member of the BRAC local village organization Respected in the community Age 20–35 Married Youngest child older than 2 years of age Have at least 10 years of education Not live near a local health facility or a large market	Supervise and support *shasthya shebikas,* provide ANC, assist with childbirth, provide newborn care, treatment for common illnesses, family planning, TB identification	2 weeks of classroom training followed by 2 weeks of field orientation and then 3 days of intensive residential training every 3 months for a 2-year period
	Family welfare assistant (FWA) (government)	Women only Have at least 10 years of schooling	Visit homes of women of reproductive age every 2 months, family planning, counselling on sexual and reproductive healthcare and HIV/AIDS	21 days of classroom training followed by on-the-job training
	Health assistant (HA) (government)	Either male or female Have at least 10 years of schooling	Provide immunizations and vitamin A supplementation, distribute ORS packets, detect and treat pneumonia, diarrhoea, malaria, and TB	21 days of training followed by on-the-job training
	Community healthcare provider (CHCP) (government)	Have at least 12 years of schooling Local resident Be able to operate a computer	Provide ANC and PNC, provide injectable contraceptives, conduct growth monitoring and nutrition education, treat minor ailments, health education and promotion	12 weeks
Brazil	*Agente comunitário de saúde* (ACS)	Selections are organized by municipalities Selection based on written test results and personal interviews	Health promotion, provide prenatal, neonatal, and child care, manage infectious diseases, link patients in need to the formal health system, provide immunizations, visit each house monthly, community mobilization	30 weeks (1,200 h) of training in three phases: a formal didactic phase (20 weeks [800 h]), a field training phase (10 weeks [400 h]) and a second formal didactic phase
Ethiopia	HEW	At least 18 years of age Have at least 10 years of schooling Preferably living in or close to the community to be served	Provide iCCM, provide family planning services (including injectable contraceptives and forearm implants), screen and refer patients with symptoms of TB, provide DOTs, and follow-up TB cases, HIV/AIDS treatment, treat malaria, treat minor illnesses, give immunizations, health promotion and disease prevention, community mobilization, home visitation	1 year of pre-service training and in-service training every 2 year
	Women’s Development Army (WDA)	Adult woman Preferably literate	Health promotion and disease prevention, support HEWs, community mobilization, serve as a role model for neighbours	52 h of training (2–3 days per week for 2–4 h per day), certificate of competency provided with successful completion of post-training assessment
Ghana	Community health officer (CHO)	Assigned by Ghana health service managers	Health education, iCCM, provide maternal, reproductive, neonatal and child healthcare, manage minor ailments	2 years (both didactic and field training)
	Community-based health planning and services (CHPS) CHV	Nominated and approved by the community Both men and women Residence in the community Can be trusted with confidential information Volunteer spirit Readiness to work under supervision Honesty	Support CHOs with referrals, transport, community mobilization, disease surveillance, and health promotion	5 days
Guatemala	Promotor de salud*	Three different scenarios: Selected by a community leader Selected by health staff Inherited from a family member	Health promotion and disease prevention	3.5 weeks (140 h)
	Guardianes de salud*		Health promotion and disease prevention, identify patients in need of referral, maintain community census	2 h per month
India	Auxiliary nurse midwife (ANM) (now called multipurpose workers-female)	Hired by the district-level health administration At least 12 years of school Female Age 17–35 years	Provide PHC, provide family planning and immunizations, screen and manage NCDs, provide elderly and palliative care	24 months followed by 3–6 weeks of skilled birth attendant training
	Anganwadi worker (AWW)	Selected by a committee of district and block-level officials Female Age 21–45 years At least a middle-school education	Provide nutritional support to mothers and children; health education	3 months
	Accredited social health activist (ASHA)	Selected by the community Female At least 10 years of education Age 25–45 years	Health promotion, home visits, household registration, promote birth at health facility, home-based newborn care	20 days within the first 18 months of joining followed by at least 15 days of additional training each year
	Village health guide (VHG)*	Politicized selection process	Care of minor acute illness	3 months
Indonesia	*Kader*	Literate Good spirit Knowledgeable about the customs and habits of the local community Local resident Friendly and sympathetic Accepted by the local community	Maternal and child healthcare, family planning, immunizations, growth monitoring of children and nutrition counselling	Less than 1 week followed by on-the-job training given by more experienced *kaders*
Iran	*Behvarz* (rural)	A formal process led by a *behvarz* recruitment committee A written examination and an interview Qualifications required: A diploma degree Both men and women eligible A native of the service area	Maternal and child healthcare, detection and management of communicable diseases and NCDs, reproductive healthcare, oral healthcare, school healthcare, treatment of minor illnesses	For those with no health background, 2 years made up of theoretical and practical coursework followed by clinical placements For those with a health-related academic degree, 6 months
	*Moraghebe-salamat* (urban)	Selection by the district health centre following examinations A native to the service area A college degree An academic degree in family health or midwifery	Maternal and child healthcare, detection and management of communicable diseases and NCDs, reproductive healthcare, oral healthcare, school healthcare, treatment of minor illnesses	Similar to *behvarz* but shorter since they have a higher level of education
Kenya	CHV	Resident of the community Has good character	Household registration and home visits, health education, iCCM, maternal and newborn care, community mobilization	13 modules split into two sections, taking around 3 months: 324 contact hours and 160 h of practical experience
Liberia	Community health assistant (CHA)	Permanent resident of the community Age between 18 and 50 years of age Fluency in the local dialect Good mobilizer and communicator Interest in health and development matters Trustworthy and respected by the community Physically, medically, mentally, and socially fit to provide the required services	Household registration and visitation, surveillance, provision of preventive, promotive, curative, rehabilitative, and palliative services, identification and management of cases of HIV, TB, neglected tropical diseases, and mental illness	8–11 days in four modules followed by several weeks of field experience
	Community health service supervisor (CHSS)	Previous training as a healthcare professional such as a nurse, certified midwife, or physician's assistant or graduate of a school of public health	Support and train CHAs, coordinate referrals, plan and coordinate outreach services	4 weeks
Madagascar	*Agent communautaire* (AC)	Elected in the village general assembly From the local community At least 18 years of age Able to read and write Have a sense of humanitarian conviction Available, motivated, and willing to volunteer Dynamic, sociable, and a good communicator Honest Both men and women are eligible	Visit homes, health promotion, participate in water, sanitation and hygiene activities, iCCM, provide family planning and TB screening	5–12 days
	*Agents communautaires de nutrition* (ACN)		Visit homes of malnourished children, growth monitoring and nutrition education, treatment of acute malnutrition, referral of malnourished children, hold cooking demonstrations	10–15 days
Malawi	CHW	Recruited from the community At least 19 years of age Able to speak English and the local language Have a minimum of a Malawi school certificate of education (MSCE) Often selected by the community they serve	iCCM, immunization, follow-up of unimmunized children, nutrition promotion, management of acute malnutrition	12 weeks (2 months of classroom training followed by 1 month of field experience)
Mozambique	*Agent polivalente elementare* (APE)	Member of the community Able to read, write, and speak in Portuguese and have basic numeracy skills Priority is given to women and to communities that are farthest from health posts	Health promotion and disease prevention, diagnosis and treatment of common illnesses, malnutrition screening, deworming	18 weeks
Myanmar	Auxiliary midwife (AMW)	Women only Living in a village without health facility or health staff Interest in health and social work Desire to stay and serve in the village after the training Middle school education In good health No more than 30 years of age Recommended by the local midwife and/or the village leader	Health education, ANC, home delivery, maternal and child healthcare, immunization, promotion of sanitation, detect and report epidemic outbreaks	6 months (3 months of theory and 3 months of practice)
	CHW	Interested in delivering healthcare and messages to the rural community Preferably younger than 35 years of age At least a middle-school education Ability to read and write the Burmese language and speak the local dialect Living in a rural area where there is no health facility	Health education, sanitation, detect epidemic outbreaks, immunization	28 days
	Malaria volunteer	Able to read and write At least primary-school level of education Recommended by the village health committee Living in a hard-to-reach, malaria-endemic village without basic health staff Not too young or too old Interested in volunteer work	Diagnose and treat malaria, health education, screen for other infectious diseases	6 days
	TB volunteer	Selected by basic health staff and township medical officer in collaboration with the community	TB screening, sputum collection and transport, accompany suspected patients for diagnosis, provide follow-up treatment support	4 days
Nepal	Female community health volunteer (FCHV)	Women aged 25–45 who are married and have children Preference given to those who are literate and living in the local community	Health education, counselling for birth preparedness, newborn care, family planning, help with outreach/immunization clinics, help with referrals to a health facility	18 days (in two phases of 9 days each)
Niger	*Agent de santé communautaire* (ASC)	At least a primary-school education Most are males	Home visits, iCCM, basic PHC, provide immunizations, provide vitamin A and ITNs	6 months with additional training following deployment
	*Relais* volunteer	Respected by community elders Both males and females are eligible	Support ACSs, home visits	Not standardized, variable in length (usually just a few days)
Nigeria	Volunteer village health worker (VVHW) and community-directed distributor (CDD)	Chosen by co-villagers	Participate in community mapping and community census; health promotion, identify pregnant women and refer them for prenatal care, promote use of ITNs and preventive malaria treatment for pregnant women, diagnose and treat malaria, treat diarrhoea and pneumonia, collect and distribute medicines to the community for onchocerciasis and other priority diseases	Training may take place in the afternoon for a few hours over the course of several weeks
	Community health extension worker (CHEW)	Formalized recruitment process Local government health department consults with community leaders Some secondary education required Resident in the local government area (not necessarily the village of service)	Follow standing orders (algorithms for clinical care) for their treatment of patients at the health facility where they are based	3 years, in formal residential school
Pakistan	Lady health worker (LHW)	Woman At least 8 years of education Between 18 and 50 years of age Resident of the area Accepted by and recommended by the community they would serve Preferably married Willing to work away from home Selection process: LHW posts are advertised; applicants are then interviewed and selected based on the above criteria by a selection committee; be recommended by a local elected official, and provide a written affidavit; then formally appointed by the district health officer	Promote and provide family planning services, ANC, treat illnesses (such as diarrhoea, malaria, pneumonia) and refer more serious cases, deworming of children, treat TB patients with DOTS	15 months in two phases: first phase consists of 3 months of classroom training; second phase consists of on-the-job training for 12 months (3 weeks of field work followed by 1 week of training per month), and 15 days of refresher training each year
Rwanda	*Binôme*	Selected by the community Criteria: Between 20 and 50 years of age At least a primary school education Willing to volunteer Resident of the village Honest, reliable, and trustworthy	Visit all households regularly, health promotion, iCCM, maternal and neonatal care, diagnosis and treatment of malaria, promote family planning and provide contraceptives, treatment of HIV and TB (DOTS), management of NCDs	3 months (on average, but not well standardized)
	*Animatrice de santé maternelle* (ASM)	Female Otherwise same as above	Visit all households regularly, health promotion, identify pregnant women and refer them for ANC and facility delivery, provide PNC and newborn care, visit pregnant women for birth preparedness, provide family planning services, screen children for malnutrition	3 months (on average, but not well standardized)
Sierra Leone	CHW	Selected jointly by the local community governance structure and the peripheral health unit Permanent resident of the community No minimum educational requirement	iCCM, disease surveillance, basic reproductive, maternal, neonatal and child care	24 days follows by 1 month of field practice
South Africa	Ward-based primary healthcare outreach team (WBPHCOT) member	Either male or female 18 years of age or older Priority given to those who are currently working as a community-based worker in another programme Residency in the community being served is preferred At least 12 years of schooling required Able to work flexible hours Mobile and able to undertake visits Legally cleared to work with children and older persons	Health promotion and disease prevention, screening and referral, rehabilitative and palliative care	1 year
Tanzania	CHW	The village health committee (VHC) manages the CHW nomination process At least 18 years of age At least a secondary school education with a pass in biology Nominated by the VHC Online application and nomination	Community mobilization, health promotion and education, identify danger signs in pregnant women and neonates and refer them for care, treat diarrhoea and pneumonia, promotion and provision of family planning services, support HIV and TB patients, provide first aid, identify mental disorders, early response and management of disease outbreaks	1 year
Thailand	Village health volunteer (VHV)	At least 18 years of age Living in the community for at least the past 6 months Literate (but no formal education requirement) Has good interpersonal relationships Generous Has at least one job	Health education and promotion (nutrition, basic medical care, sanitation and clean water, maternal and child health and family planning, immunizations, dental health, mental health, HIV prevention and control	43 h of classroom work with ongoing in-service training
Uganda	Village health team (VHT)	Selected by community members and local leaders Respected and trustworthy, good listener, dependable, approachable Preferably previous experience in volunteering At least 18 years of age Able to read and write and speak the local language Be a good communicator and community mobilizer	Home visits, health education and promotion, promote good nutritional practices and safe water use, promote utilization of maternal and child health services and family planning, treat childhood malaria, pneumonia, and diarrhoea, and support drug distribution for endemic diseases	5 days (+ an additional 6 days for those who learn iCCM
Zambia	Community Health Assistant (CHA)	Completion of 12 years of school and 2 “O” levels (one should be in English) 18–38 years of age Living in the recruitment catchment area for at least 6 months Endorsed by the Neighbourhood Health Committee and traditional leaders Passed a personal interview with a panel of Neighbourhood Health Committee members, health centre staff, and a member of the district health office Have previous experience with community health work	Health education and promotion, provision of basic curative services at a health post, iCCM, mental health counselling, pregnancy and HIV testing, promote family planning and provide oral contraceptives, take blood pressure to identify patients with hypertension, measure urine glucose to identify patients with diabetes, provide first aid and palliative care, identify disease outbreaks, promote use of ITNs, promote clean water, water purification, latrines (and support community-led total sanitation efforts), and food hygiene, identify patients in need of referral, provide deworming medicine, provide immunizations, map the catchment area	1 year (36 weeks of formal classroom training followed by 16 weeks of field training)
Zimbabwe	Village heath worker (VHW)	At least 25 years of age Married resident of the village Able to read and write Possesses strong communication skills Respected in the community Interested in health and development issues Willing to work in the community on a volunteer basis Able to keep personal health information confidential	Health promotion and prevention, promote immunizations and support immunization campaigns, provide DOTS for TB patients, provision of basic curative services, support patients with chronic conditions, malaria prophylaxis for pregnant women and children, growth monitoring, promote HIV voluntary counselling and testing	5 months

All of the information in this table has been abstracted from the 2020 book, *Health for the People* [34]. Each of the 29 case studies in this book has a section on selection and training of CHWs. All of the CHW cadres described here are working in the public sector except for the *shasthya shebikas* and *shasthya kormis* in Bangladesh, who work under the auspices of an NGO, BRAC, and operate independently of—though in cooperation and in collaboration with—the public sector. The CHWs in Afghanistan are trained and hired by NGOs who receive government contracts that are set the guidelines for their training. Similarly, the nutrition CHWs in Madagascar, *agents communautaires de nutrition* (ACNs), work for NGOs who are contracted by the government. The book does not provide detailed or consistent information about the process of selection of CHWs. Further details about the roles and tasks of each CHW cadre as well as further information about how the cases were selected can be obtained from the book, which is available at: https://pdf.usaid.gov/pdf_docs/PA00WKKN.pdf

*ANC* antenatal care, *CHV* community health volunteer, *DOTS* directly observed therapy, short course (for TB), *HEW* health extension worker, *HIS* health information system, *iCCM* integrated community case management (of childhood illness), *ITN* insecticide-treated bed net, *NGO* nongovernmental organization, *NCDs* noncommunicable diseases, *ORS* oral rehydration salts, *PHC* primary healthcare, *PNC* postnatal care

*not currently functioning

Training of other health workers to more effectively engage and collaborate with and support CHWs has been limited, with many programmes providing no explicit training and or support to other cadres [38]. While issues and challenges related to supervising CHWs (both for clinicians and managers who supervise CHWs as well as for CHWs who supervise other CHWs) have been explored in South Africa, Tanzania, and other contexts [39, 40], there is limited documentation in the academic literature of any training programmes or similar support services that have been developed and utilized to prepare and support supervisors in these important roles [41]. Technology has also been held up as a solution to CHW supervision challenges—some of which it can alleviate, such as timely communication and access to current information [42–44]—but it is not a replacement for key relational aspects that are critical to CHW motivation and effectiveness, including valuing their unique contributions and establishing appropriate incentive structures. (See Paper 7 on supervision [28] and Paper 8 on motivation [29] in this series for further discussion of these issues.)

### Training-related priorities for the future of CHW programme scale and sustainability

This section is organized around a set of priority considerations related to training for CHW programmes going forward. We have organized these around three overall themes: (1) professionalization, (2) quality and performance, and (3) scale-up. We will describe each and provide some background on the experience to date and examples for each one, and then describe some challenges and opportunities that can help CHW programmes navigate each going forward.

### Implications of the recent professionalization of training for CHWs

Over time, the engagement of health systems with communities has increased [45–47]. Communities expect a more professionalized and capable health system, including professionalized CHWs [48–50]. Professionalization entails the processes, including for CHWs, whereby an occupation becomes established as a recognized line of work, with formal entry-level training and established remuneration. CHWs and health systems have been evolving to meet this demand by expanding training programmes, establishing standards, improving remuneration, and ensuring that CHWs are recognized by communities and other professionals in the health systems [51]. Two major pressures driving professionalization are rising expectations by communities for (1) a trained healthcare worker to be available on a full-time basis to provide clinical services and advice at the community level and (2) referral to and counter-referral from higher-level healthcare services [52]. Generally, the shifts in roles have included expansion of services to include prevention and treatment of NCDs, mental health, data management and disease surveillance, as well as expanded clinical care roles; further details about the changes in CHW roles were described earlier in the Context section as well.

At the same time, socioeconomic inequities persist and are even increasing regarding who can afford what kinds of care [53]. All of this means that CHWs require additional training in order to provide the level and complexity of clinical care and authoritative guidance and coordination to navigate the rest of the health system that is needed to support improved health status at the community level—particularly for remote and underserved populations—and to meet community expectations [53–55]. Numerous factors, such as distance, lack of money for transport, and medical fees, are clearly demarcating sections of the community who do not have access to health facility-based care [56–58]. The inability of some people to travel to a facility for essential services has led to further task sharing and shifting to CHWs in some countries, though not all.

Pressures on CHWs are compounded by the continued shortage of other key cadres of PHC workers, including nurses and primary care physicians in many contexts, particularly in rural and disenfranchised populations [9, 59–64]. In addition to social and cultural drivers of shifts in the roles and expectations of CHWs, changing healthcare needs of populations caused by ongoing demographic and epidemiologic transitions are also driving the burden of disease and what is needed to sustain and improve the health of communities [65, 66]. Further, CHWs also need to be considered as full partners and as adult learners, and they need to have a role in designing and engaging in their training experiences in order to have strong ownership of their roles and carry forward with their essential services, even during times of transition and challenge in the systems where they work. Health systems must evolve, and therefore so must the package of health services that CHWs are expected to provide. Of course, these services are often organized in collaboration with other providers, and some countries are also seeking to expand the number of workers in other cadres of workers such as doctors and nurses. As new priorities arise, such as NCDs, mental health, and newborn care, stronger PHC teams that include a formal role for CHWs that are able to provide valued services directly to communities and can also employ referral strategies are needed [67–69]. New services and protocols will need to be added to packages of essential services provided by CHWs at the household level, and trainings will be needed to conduct this work effectively.

Increasingly, health programmes are training and using higher-level CHWs. Some of these CHWs have a significant community engagement focus (such as the HEWs in Ethiopia), while others (such as the CHCPs in Bangladesh) spend most or all of their time based at a health post. Even in instances where health systems rely on volunteer CHVs to promote healthy behaviours and to link their communities with the health system, these volunteers often include more highly educated youth who are seeking professional opportunities to secure more stable and better-paying work as their careers progress [52].

As more highly educated CHWs are recruited and are trained more extensively, new challenges will arise. For instance, it may become more difficult to recruit CHWs from the communities they will service, and it may also become more difficult to retain them there [70]. As training durations increase, CHWs will need to invest more time in school and may also have to travel to official and approved training institutes that can manage and staff these longer, more intensive training programmes. These more academically oriented training venues provide opportunities for trainees to be exposed to other health professionals and gain confidence working in different settings, but the training may also become less connected and less relevant to the local contexts where CHWs will work.

The single strong recommendation related to training in the WHO guideline proposes that marital status *not* be a criterion for selecting CHWs for training and deployment. The guidelines call for selection of candidates who are first approved by and acceptable to the community where they will be working and who have the minimum level of education appropriate to the tasks for which they will be trained and with the necessary cognitive abilities, integrity, motivation, interpersonal skills, commitment to community service, and a public service ethos. Community participation in the recruitment and selection process not only enhances the appropriateness of the persons selected, but it also “enables a dialogue between community members and health organizations, helping them understand local issues” [37].

As the package of services expands, the training of CHWs needs to become more professionalized and better integrated into the health system [71, 72]. If not, CHWs can become overworked, burned out, and frustrated [73]. Questions then arise about the benefits and polyvalent versus specialized CHWs to support specific subpopulations or to provide a more targeted set of services, such as screening and support for chronic disease management [3, 6]. (This is an issue we discuss elsewhere in this series [11].) There is no simple way out of this dilemma, but as more countries and health systems shift towards handling higher burdens of chronic disease, testing different approaches to integrated versus more specialized roles for CHWs will help guide policy and best practices.

At the same time, as the demand for highly skilled and professionalized CHWs is growing, appropriate and invaluable roles remain for lower-level CHWs, often working at the neighbourhood level with a smaller scope of responsibilities and on a voluntary basis. These CHWs, such as the Women’s Development Army (WDA) members in Ethiopia (see Box 1), are able to have frequent contact with their neighbours and focus on promoting healthy household behaviours, identifying pregnant women, and linking households to higher levels of care when needed. In addition, these lower-level CHWs are directly connected to, trusted by, and have first-hand understanding of the poorest and otherwise socially marginalized sections of communities with the most limited access to facilities. Such volunteer CHWs are often trained and supervised by more professionalized cadres of CHWs, and they rely on job aids such as flip charts and culturally appropriate approaches such as songs, stories, and role playing to share health information and to demonstrate healthy behaviours [74, 75]. In addition, collaborations between different cadres of CHWs can support the effective provision of services such as home-based neonatal care (HBNC) as is done by the NGO SEARCH in Maharashtra, India, as well as in other programmes [76–82]. Finally, female CHWs, such as the female community health volunteers (FCHVs) in Nepal [83–85] and the volunteer female CHWs in Rwanda (the *animatrice de santé maternelle* [ASM] and the female member of a pair of CHWs called a *binôme*) [86] have the ability to promote women’s empowerment and social solidarity as well as to address the social determinants of health in ways that professionalized CHWs will not have time to do.

In our haste and under various societal pressures to modernize and professionalize large-scale CHW programmes, we must not forget the value and the unique contributions that can be made by community-embedded, sustainable, and trusted volunteers and workers who have no intention to leave their community.

**Table:** 

**Key message 1**
Professionalism of CHW training does not negate the invaluable contributions of volunteers working in community settings to improve the health of populations.

Box 1. Training CHWs in Ethiopia’s health extension programme In 2003, the Government of Ethiopia launched its Health Extension Programme (HEP) as part of an accelerated PHC expansion. The CHW cadre that was introduced at that time. HEWs, had to completed 10 years of formal schooling followed by a 1-year course of instruction along with field training or an apprenticeship. The number of training packages also increased from 16 to 18 as a result of adding modules on NCDs, mental health, and neonatal care. Most trainings for HEWs—including on-the-job trainings and integrated refresher trainings, including specific trainings on giving new vaccines and placing long-acting contraceptive implants—are provided by the public sector.

Ethiopia’s HEP is an example of a large-scale and professionalized dual-cadre CHW programme [34]. The HEP arose in response to the realization of the Ethiopian government in the late 1990s and early 2000s that healthy household behaviours were not common, access to basic and essential services was inadequate, and the shortage of health workers, particularly in rural areas, were all responsible for Ethiopia having some of the poorest health indicators globally [87]. As the second-most populous country in Africa, now around 110 million people living in diverse climates and a range of economic bases and cultural traditions, Ethiopia needed a health system that could respond to these needs not only for dispersed rural agrarian populations (which comprised the great majority of the country) but also for those in remote tribal nomadic populations as well as in rapidly growing urban areas [88].

The decision to introduce a well-educated and highly skilled female CHW, the HEWs, arose partly in response to the growing literacy levels in the population and the related demand for quality health services, particularly curative and disease prevention services. The training of HEWs began in 2003, and now there are approximately 40,000 HEWs throughout the country. A pair of females HEWs work together to staff a health post which serves approximately 1000 households (or 5000 people). The HEW divides her time between the health post and the community.

In 2011, the government recognized that HEWs alone would not be able to achieve all of the desired changes related to health that Ethiopia planned. Therefore, they converted the other existing volunteer CHWs throughout the country into the voluntary WDA. The WDA consists of a network of neighbouring households to increase the efficiency and reach of HEWs. The WDA leaders receive 52 h of training primarily on health promotion and community mobilization and are tested on their skills at the end of the training (see Table 1 for further information). These networks are organized in the community with one WDA volunteer for every 5–6 households. Every five WDA volunteers select a team leader (referred to as the “1 to 5 Network leader”) Each team of five WDA volunteer women with their WDA leader then works with 30 households (the “1 to 30 Network”). The WDA leaders receive training on key health actions consistent with the HEWs’ scope of work and are supported and supervised by the HEWs as well as by the health centre and district (woreda) health office staff. The HEWs also provide informal training and support.

Initially, the emphasis was on reaching rural agrarian regions of the country that lacked access to health services, and the programme was adapted and scaled into pastoralist regions in 2007 and into urban areas in 2009 [34].

**The training programme for HEWs**

Candidates who had completed a tenth-grade education are recruited. Community members and representatives from district health offices, from the offices of education and women’s affairs, and from offices of other relevant sectors select the HEWs. Preference in selection is given to candidates who are from or close to the same community (*kebele*) they would serve. However, if suitably educated candidates do not exist in the *kebele*, preference is given to candidates from a neighbouring *kebele* who met the selection criteria. Hence, in virtually all cases, HEWs understand the language, culture, lifestyle, behaviours, and practices of the local community and are thus able to work effectively with the communities in their catchment areas.

HEWs receive 1 year of pre-service training from a technical vocational training institute or from a health college. HEW training is conducted in collaboration with the Federal Ministry of Health (FMOH) and the Ministry of Education. HEW training includes didactic and clinical training in modules on (1) family health services, (2) disease prevention and control, (3) hygiene and environmental sanitation, (4) health education and communication, and (5) documentation and health information. HEWs also receive in-service integrated refresher trainings to build on their initial training and develop new competencies. Most HEWs have undergone multiple continuing education trainings on communicable diseases and reproductive health, among other subjects. There was, however, little coordination among these trainings, leading to duplication of efforts and wastage of time and resources. There is widespread agreement that the quality of training provided to HEWs needs improvement. In order to provide standardized and harmonized in-service trainings for HEWs, the FMOH developed its integrated refresher training approach.

HEWs have expressed a desire for additional training on other topics such as basic nursing care, attending deliveries, and treatment of children with common childhood diseases [89]. Since 2010, HEWs have received trainings on iCCM, enabling them to manage life-threatening childhood illnesses at the community level, including pneumonia, diarrhoea, malaria, malnutrition, measles, and ear infections [90]. The standard curriculum content has expanded in recent years to include mental health and NCDs as population health needs evolve. In addition, refresher training is provided every 2 years (though sometimes may not regularly be offered due to various constraints) in order to update service delivery protocols and provide HEWs with additional skills and knowledge; the importance of this training is recognized, and further efforts are underway with the integrated refresher training approach to avoid duplication of trainings and improve the quality of the offerings. Almost half (48%) of HEWs have now received sufficient additional training to enable them to move to a higher grade (level IV) and earn a better remuneration package [91]. However, 25% of HEWs have still not completed their full formal initial certification examination [91].

**Interprofessional training**

In parallel to the establishment of the HEW cadre, Ethiopia has also trained medical students, midwives, and health officers to work in the health centres and primary hospitals. Part of the recent curriculum includes how to collaborate with the HEWs and work within the PHC level of the health system, including managing communications and referral and counter-referral processes with the HEW-staffed health posts and supervising the HEWs [34].

**Impact**

Developing and sustaining the HEW cadre has been an ongoing commitment and investment by Ethiopia’s government. Despite early scepticism about the ability of HEWs to demonstrate the bold impacts that Ethiopia aimed to achieve as well as the feasibility of securing adequate funds to support the programme [88], the period since the establishment of the HEP has shown a number of positive impacts for Ethiopia. Steep declines in under-five mortality and maternal mortality have taken place, and the burden of HIV, TB, and malaria has also been reduced [34]. HEWs have contributed significantly to these positive health changes due to their ability to improve access to many basic and essential services [88]. These positive results have reinforced the value of the programme and have garnered ongoing support to sustain and improve it [88].

The HEP has been lauded as an exemplary model for how CHWs can contribute to well-functioning PHC systems and contribute to improved health outcomes [87]. Since then, political commitment, coordination among many development partners, and a focus on continually improving the HEP has led to a nationally scaled, highly active, and fully embedded programme that supports the rest of Ethiopia’s health system [87].

### Assuring quality and performance selection of CHWs, job-design, training approaches, job-aids, task shifting, and strategies for achieving high performance and quality

The responses of health systems in low- and middle-income countries to the challenges and opportunities they face have important implications for the roles and training of CHWs and their relationships with health facility staff—both clinicians and managers. CHWs have contributed to the progress made by health systems in many countries. However, in order to continue this progress, greater equity in service provision and improved overall CHW programme performance and quality will be required. This calls for new strategies, including a greater emphasis on integration of community-based activities with facilities.

The WHO guideline on CHWs does indicate a general consensus that inadequate training will leave CHWs unprepared for their role and most certainly adversely affect their level of motivation and commitment, not to mention the quality of their work [37]. The length of training should be based on the scope of work and competencies required, the trainees’ pre-existing level of knowledge and skills, and other contextual factors. Most of all, the duration of training should be sufficient to ensure that the desired competencies and expertise are achieved but also that they are feasible, acceptable, and affordable [37]. However, there is not a strong evidence base upon which to make decisions regarding what kind of trainings and what lengths are most effective.

### Selection of CHWs

Some programmes, like the Afghanistan programme, deliberately have both male and female CHWs in a community and encourage selection of men and women who are related so that they can work together [92]. The selection of women is sometimes constrained by educational requirements, leading to an under-representation of women in the CHW workforce. The effectiveness of male CHWs can be restricted by their gender, particularly in discussing issues related to sex and reproduction [93]. Examples exist of successful programmes that have selected CHWs from among the poor [94]. Even when CHWs are selected from among the poorer members of a community, other factors can still impede success.

### Dual-cadre models

The emerging role for CHWs is that of a multipurpose, professionally trained, and salaried worker who brings services closer to the community and coordinates with other health professionals when patients require additional care. Increasingly, these more highly trained CHWs are complemented by village volunteers who serve a smaller number of households. For instance, in Ethiopia, the HEW, who serves about 500 households or 2500 people, WDA volunteer leaders who coordinate 30 or so households [95]. Another example of the dual-cadre approach is in the BRAC CHW programme (see Box 2), where a higher-level, salaried *shasthya kormi,* who is herself a CHW, supports the lower-level *shasthya shebika.* When a *shasthya shebika* identifies a new pregnancy or birth, she calls on her *shasthya kormi* to come to the home to provide care and education, which are beyond the scope of work of the *shasthya shebika* [96, 97].

### Training approaches

Training for CHWs has evolved over the years and has recently expanded in terms of scope and modalities. More emphasis has also been placed on application of knowledge through simulations, supervised practice, being able to demonstrate skills under observation, and peer assessments. In addition, attention to how CHWs can most effectively learn has taken on a higher priority in recent years. Increased focus on storytelling, case-based learning, and peer learning and teaching have helped align CHW training with cultural norms for sharing information with each other.

Teaching problem-solving skills and other more complex tasks and responsibilities has required other training approaches beyond basic classroom lectures and memorization. Training is also needed on how to interact with families and communities and how to handle challenging situations that they will certainly face: conflict within and between families (including gender-based violence), lack of medicines and supplies, lack of supervision, and how to deal with health problems for which they do not have adequate training (e.g., severely ill patients, mental health problems, social conflicts) but which communities expect them to be able to address. Finally, and critically, trainees need guidance on how to deal with stress and burnout arising from these issues as well as from the common issues of being stigmatized and overworked.

The WHO guideline on CHWs recommended that training encompass at a minimum: (1) promotive and preventive services, (2) identification of family health and social needs, and risk factors for them, (3) integration within the wider healthcare system (referral, collaboration with other health workers, patient tracing, disease surveillance, monitoring and data collection, and analysis and use of data), (4) social and environmental determinants of health, (5) provision of psychosocial support, (6) strengthening of interpersonal skills related to confidentiality, communication, community engagement, and mobilization, and (7) personal safety [37]. The guideline concluded that training for diagnosis, treatment, and care be added to this basic set of domains when these are a part of the expected role of the CHW and when regulations on scope of practice are not prohibitive [37].

Although the level of evidence was low, the WHO guideline for CHWs also made a conditional recommendation that the content of training be a balance of theory-focused knowledge and practice-focused skills, including supervised practical experience [37]. The WHO guideline recommends that every effort should be made to provide training in or near the community and with learning methods in a language appropriate for the trainees. When possible, interprofessional training (with other types of health workers) should be encouraged, as well as the creation of a positive training environment. E-learning can supplement other modalities of training and is particularly appropriate for follow-up and refresher training [37]. This has been found in Ethiopia and Bangladesh (see Boxes 1 and 2), and has also been found to be cost effective [98] and to support knowledge exchange between CHWs [99].

An important but unexplored field of inquiry concerns the pros and cons of what kind of organization is best suited to provide training for CHWs and at what level in the healthcare or educational system. In some countries, universities and ministries of education take responsibility for training CHWs, while in others, the training is provided by the MOH or by technical institutes within the MOH. The level of centralization or decentralization of training is also a consideration, and how far away from home CHWs have to travel to attend training is an important issue that training programmes have to face.

### Task shifting

Since the initial development of the concept of the integrated continuum of care in maternal, neonatal, and child health (MNCH) was developed, the scope of activities recommended for traditional birth attendants (TBAs) and CHWs has been expanded beyond health promotion. This is particularly important for implementation in populations in which access to facility-based healthcare is limited. These recommendations include distribution of nutrition supplements to pregnant women, the provision of bed nets and monthly intermittent presumptive treatment (IPT) of malaria, the administration of misoprostol to prevent postpartum haemorrhage for home deliveries, and the provision of injectable contraceptives [100]. There is accumulating experience with community-based implementation of nutrition supplementation [101, 102], malaria control [103, 104], misoprostol [105, 106], and injectable contraceptives [107, 108]. There is also growing support for the benefits of training of TBAs in clean delivery and cord care, immediate newborn care and referral of complications [109], newborn resuscitation [110], and organized postnatal home visits and management of neonatal infections [111, 112].

### Job aids

Professionalized CHWs are also often expected to provide training to other volunteer cadres, and most CHWs are expected to provide health education to community members. What sorts of visual aids and tools are most helpful for this to be done effectively? For example, the *shasthya kormis* in the BRAC programme in Bangladesh have recently started this, using computer tablets for this purpose (see Box 2). Tablets are emerging as an important resource for showing key health messages through drawings, thereby eliminating the need for heavy paper flip charts.

*Shasthya kormis* are now also using tablets to record census data as well as case records of pregnant women (see Box 2). An Android-based mHealth system was used in a similar way to survey a population of two million individuals in Western Kenya for TB and HIV. Several hundred government-trained CHWs offered home-based counselling and testing for HIV along with sputum collection from individuals with symptoms of TB. Data were downloaded to a central server and then deleted from the CHWs’ phones for reasons of confidentiality. Collated information was provided to the clinics that were supervising specific communities. The CHWs found the system easy to use and preferable to the previous paper-and-pen alternatives. The system was also found to be more cost-effective than the pen-and-paper system [113].

### Strategies for improving performance and quality care

The comprehensive review of the evidence regarding the effectiveness of community-based PHC in improving MNCH [114] identified four community-based strategies used by effective projects: a) treatment and/or referral of sick children by parents or CHWs, b) routine systematic visitation of all homes, c) facilitator-led participatory women’s groups, and d) provision of outreach services by facility-based mobile health teams [115]. Most importantly, most (78%) of the studies included in the review that measured equity effects of community-based programmes reported that these effects were “pro-equitable”, meaning that the effect of the programme was more favourable for the most disadvantaged segment of the population served than for the rest [116].

Participatory women’s groups have been particularly successful in changing complex sets of behaviours that are embedded in cultural belief systems. Recent experience has demonstrated that it is in the realm of pregnancy, childbirth, and newborn care that participatory women’s groups have been effective. Both maternal and neonatal mortality rates have been reduced in communities, in proportion to the numbers of pregnant women participating [117]. Four mechanisms seem to explain the impact of the women’s groups. The first is that during their pregnancy women learn about appropriate care and how to prevent and manage problems. This process is enhanced by discussion and learning from each other. The second is the development of confidence. This is particularly important when dealing with mothers-in-law or other authority figures in the community who are advocating traditional beliefs and practices or resisting healthy practices. The third is the dissemination of information to others in the community. In addition to the formal community meetings, there was continued informal sharing among family members and neighbours. In one project in Malawi, there was more intentional home visiting to share information. Finally, the fourth mechanism is building the community’s capacity to take action [118, 119].

Training needs to provide all stakeholders, not just CHW trainees, with exposure to evidence-based new approaches such as those described here so that they can at least begin to sense the potential of modifying their strategies to improve performance and quality of the CHW programme.

**Table:** 

**Key message 2**
Assuring quality of CHW training does not just entail more training, but rather ensuring that training is relevant, that associated job aids are available, and that tasks and expectations are aligned with the training provided.

Box 2. The BRAC approach to training and supervision of its CHWs BRAC is the largest NGO in the world and perhaps the most renowned as well, having been repeatedly designated as the best NGO in the world by NGO Advisor [120]. Its programmes reach 130 million people in 11 different countries [121]. BRAC began in Bangladesh in 1972, where its programmes now reach 110 million people [122]. BRAC’s programmes and approaches that have been effective in Bangladesh are adapted for implementation in other countries. BRAC’s integrated approach to the development of the most marginalized community families emphasizes the empowerment of women, the promotion of diversified and sustainable livelihoods, supported by microfinance and improved access to essential services in education, health, water, and sanitation.

BRAC’s health programmes are entirely community-based. Links for referrals, training, and support are made locally with government or other NGO programmes and hospitals. BRAC has three CHW cadres: *shasthya shebikas, shasthya kormis,* and programme assistants [97]. *Shasthya shebikas* are women who are part-time volunteers, and *shasthya kormis* are full-time salaried workers. A second programme in NCDs is implemented by a programme assistant. *Shasthya shebikas* are women selected from and by their communities and have at least an eighth-grade education. They are responsible for carrying out monthly home visits to 300–400 households in their communities. The home visits are guided and monitored by use of a pictorial register, in which the *shasthya shebika* keeps a tally of her activities and the services she provides. Her main tasks are health promotion, using posters and flip charts when available, promoting use of MNCH services, identification of pregnancies, treatment of minor illnesses, and sale of health-related commodities (oral contraceptives, condoms, essential medications, iodized salt, birthing kits, sanitary napkins, and even vegetable seeds). BRAC provides start-up money for purchasing the health commodities that the *shasthya shebika* then sells. BRAC also provides performance incentives for identifying pregnant women who will need care. Until recently, there were more than 100,000 *shasthya shebikas.* BRAC has reformulated the programme to double the catchment area for each *shasthya shebika* and provide a better financial remuneration package based partly on performance incentives (for identifying pregnancies and referring patients in need of higher levels of care) as well as on the traditional sale of health-related commodities. There is a 7–10% annual turnover of the *shasthya shebikas.*

The *shasthya kormis* are also women, selected from their communities who have at least a tenth-grade education. They receive the same initial training as the *shasthya shebikas* and then later receive additional training in prenatal care and postnatal care from government institutes. About one half (2000) of *shasthya kormis* have obtained 6 months of formal training at a government institute in obstetrics, and they perform home deliveries. They supervise 10–12 *shasthya shebikas* while carrying out home visits together twice a month to women and children needing MNCH services. The *shasthya kormis* maintain and update household demographic registers in all these communities twice each year and report births and deaths. They provide education to families about MNCH and nutrition, and they provide ANC and PNC to women and their babies, and also family planning services. The *shasthya kormi* keeps a record of the progress of the pregnancy and the newborn in her own MNCH register, but also in the client’s home-based MNCH handbook, which also has relevant pictorial health messages for the family. The *shasthya kormis* are now also promoting awareness of common gynecological problems and teaching women to self-examine for breast lumps. They are now expanding their activities to specifically address health issues of adolescents.

The CHW cadre of programme assistants has recently been created to organize and implement community-based outreach clinics to screen for hypertension, diabetes, and common eye problems (and provide eyeglasses for those with presbyopia). Each programme assistant works with 50–60 *shasthya shebikas.* They receive the same initial training as *shasthya shebikas* and *shasthya kormis*, but then later receive short technical courses on the interventions that they provide. They hold 11–12 clinics every month, and each community is visited twice a year in collaboration with the *shasthya shebika*. At present, there are 631 programme assistants.

**BRAC’s training programme**

The principles and approaches of the training programmes for each of the three cadres are the same. Here we highlight seven important characteristics.

1. *Incremental*
  The full training of a *shasthya kormi* takes place over the course of 2 years. For the *shasthya shebika* and programme assistants, it is shorter. The basic structure of the training programmes is made up of an introductory training of about 2 weeks, followed by monthly 1-day refresher training meetings. In addition, there are 3-day training meetings, frequently residential, for more intensive technical training. These occur regularly every 3 months for the *shasthya kormis*, but less frequently for the *shasthya shebikas*. This process allows for a progressive accumulation of skills that are practiced and supervised in the work setting, and reinforced as necessary in the refresher meetings, before another set of skills is added. Training needs for individual CHWs are identified in part by an independent quality control group that is out in the community observing the work of BRAC CHWs.
2. *Competency-oriented*
  The emphasis on technical and communication competencies means that adequate time is allocated to supervised practical experience in clinical settings and in the context of home visits and women’s group meetings. Training supervision in each setting is provided by both training staff and experienced programme supervisory staff.
3. *Use of job aids*
  Quality assurance and complete community coverage is assisted by the use of job aids. Each of the CHWs uses an appropriate register that requires the recording of specific observations or services provided. The home-based MNCH handbook serves as a parallel performance guide and record of the progress of a women’s pregnancy. To maintain the standards of health behaviour change communication, visual aids have been in use for key sets of MNCH messages.
4. *Professional trainers*
  The key training staff are all full-time and committed to training, forming a pool of master trainers. Most are health professionals or community programme support staff, who have demonstrated technical, social, and communication skills in BRAC community programmes over a number of years. They are then introduced to BRAC’s training philosophy and methods.
5. *Convenience- and cost-sensitive*
  In the past, training courses for the *shasthya shebikas* were often residential and held in BRAC offices. Recently, the emphasis has moved to doing the introductory training courses in a house or facility to which the women can conveniently travel each day. The more expensive and less convenient residential training is kept for essential clinical training.
6. *Modernizing*
  Digital devices are being used increasingly. Digital registers on tablets are now being used for updating household demographic data and for the MNCH clinical registers used by the *shasthya kormis*. In addition, they are now using some video health education materials during home visits or meetings with groups of women.
  The training programme is now making more use of both generally available digital materials as well as digital training materials prepared by and for BRAC using an app call Dishari, which self-monitors learning and identifies areas where further learning is needed, thereby reducing costs of training. Both written and video materials are used for refresher training, together with pre- and post-testing. This provides immediate feedback to the individual health worker, but also provides feedback to BRAC of the topics that are less well understood and, therefore, need extra reinforcing. This has been particularly useful in responding to the COVID-19 pandemic. Since the *shasthya kormis* all have tablets, they have received the same online training for the pandemic response.
  The capacity to provide online training for *shasthya kormis* reduces the cost of training considerably, and it makes it possible to readily include videos as well. Following the completion of a course, *shasthya kormis* take an exam, and the results are maintained in a database with the results available for each individual. Special online trainings are being developed to address weaknesses identified for each individual *shasthya kormi.*
7. *Individualized*

Records are kept of the training courses and experiences of each CHW. This has been a paper record in the past, but is now being digitized. The availability of individual training records means that refresher courses and training in new skills can be planned appropriately to reflect the different stages of training and experience of each CHW.

Table 2 below provides a summary of the evolution of the BRAC CHW programme over the past 60 years and how its training of CHWs evolved in the process. The transition from a focus on maternal and child health, nutrition, family planning, and WASH (water, sanitation, and hygiene) to a broader focus is readily apparent. Now included are NCDs, eye care (including provision of glasses for the elderly), psychosocial counselling, and even climate change mitigation. mHealth innovations are being introduced that make possible real-time demographic and management information. The professionalization of the CHW cadres is also readily apparent, with longer initial training and also more intense and higher-quality continuing education. BRAC’s impact has been assessed by many programme evaluations carried out by its renowned Research and Evaluation Division.

**Table 2:** Evolution of BRAC’s CHW programme over 60 years (1970–2019)

Characteristic	Evolution over time				
Time period	1970–1979	1980–1999	2000–2009	2010–2019	2020–2029
Socioeconomic environment	Extremely poor, with a low literacy level and poor communications infrastructure	Poor but literacy and communication improving	Poor with acceptable literacy and communication networks	Low-income country but developing and urbanizing, with increased access to digital networks	Developing country that has achieved status as a lower middle-income country in process to become an upper middle-income country, population better educated, older, more urbanized, and well connected to digital communications
Stage of development of CHW programme	Experimental, pilot	Embryonic	Expanding	Maturing	Entrepreneurial
Type of CHW envisioned	Empowered woman	Educator and mobilizer	Healthcare service provider	Healthcare service provider who is sustained through cost recovery	Business women who is a service provider who earns the money herself needed to sustain herself
Objective of CHW programme	Empowerment of women (CHWs with access to knowledge, social recognition, and financial inclusion)	Improved child health	Improved access to health services and health information	Improved resilience of community in meeting its health needs	Improved access to quality healthcare through a digitally enabled community-based healthcare system
Nomenclature for CHW cadres	Paramedic, lady family planning officer	Oral therapy extension worker, *shebok shebika*	*Shasthya shebika, shasthya kormi,* nutrition promoter	*Shasthya shebika, shasthya kormi,* programme assistant, mid-level ophthalmic paramedic, midwife, skilled birth attendant	*Shasthya shebika, shasthya kormi,* programme assistant, mid-level ophthalmic paramedic, midwife, para-psychosocial counsellor
Tasks	Provision of over-the-counter drugs and family planning commodities at the home	Education and mobilization for diarrhoea prevention and treatment, immunization, vitamin A, demand creation for public health services, maternal and adolescent health, and communicable diseases	Promotion of positive health behavior, creation of demand for public and BRAC services, provision of services free of cost at households and outreach points, paper-based data collection	Promotion and demand creation, service provision with service fees, health centre-based service provision, introduction of digital data collection	Promotion, demand creation, service provision, psychosocial counselling, digital real-time recording of demographic and management data
Scope of services	Provision of family planning commodities, treatment of common ailments	Child health (prevention and treatment of diarrhoea, child survival interventions); WASH interventions; ANC, safe delivery, and PNC; adolescent family life education, nutrition supplementation	RMNCH; communicable diseases (TB, malaria); child feeding, dietary diversification, and micronutrient supplementation), eye care	RMNCH, NCDs, nutrition, eye care	RMNCH, NCDs, nutrition, eye care, mental health, early childhood development, food safety, climate change mitigation
Training duration	2 months for basic training with monthly refresher training	1 month of basic training with monthly refresher training	18 days of basic training with monthly refresher training, provision of new knowledge periodically	Basic training over a 2-year period (initial 3 weeks of basic training followed by 3 days of basic training every 3 months for 2 years, and then monthly problem-based refresher training)	Same as in 2010–2019
Training content	Family planning, common ailments	Child health (prevention and treatment of diarrhoea, child survival interventions); WASH interventions; ANC, safe delivery, and PNC; adolescent family life education, nutrition supplementation	RMNCH, communicable diseases (TB, malaria), nutrition and IYCF (promotion of appropriate infant and young child feeding), dietary diversification, and micronutrients; eye care	RMNCH, NCDs, nutrition, eye care	RMNCH, NCDs, nutrition, eye care, mental health, early childhood development, food safety, climate change mitigation
Training methodology	Pedagogy (face-to-face lecture-type learning)	Pedagogy (face-to-face, lecture-type learning) using printed materials, flip charts, and posters	Combination of pedagogy with participatory adult learning using audiovisual aids, simulation games, field placements, and clinical training in health facilities	Subject-based training with lengthy courses of up to 1 year in not duration; specialized training institutes are contracted to give courses	After in-person basic training, digital training is provided depending on skill needs; self-learning provided through a digital platform
Impact	Public health orientation started in the country	Reduced numbers of diarrhoea deaths, reduced night blindness, improved child vaccination coverage, improved ANC coverage	Improved CPR, reduced number of child and maternal deaths, increased case identification and treatment completion of TB, reduced severe malnutrition among children	Use of clinical contraception improved, reduced number of child and maternal deaths, stunting reduced, improved access to treatment for communicable diseases and NCDs, improved access to eye glasses to correct refractive errors and to cataract surgery	Access to quality services improved

*ANC* antenatal care, *CPR* contraceptive prevalence rate,*IYCF* infant and young child feeding, *NCDs* noncommunicable diseases, *PNC* postnatal care, *RMNCH* reproductive, maternal, neonatal, and child health, *WASH* water, sanitation and hygiene

## Discussion

Scaling up of CHW programmes includes expanding population coverage, of course, but it can also include improving quality and adding new components to existing programmes [123, 124]. Scale-up often builds on the experience of a pilot phase or on the experience of more widespread programme implementation. As such, when designing the adaptation or expansion of a CHW programme, it is important to consider who all the stakeholders will be, how their roles will change or grow in the new programme, and what kinds of training needs may arise for them. Secondly, considering who the best-placed trainers and mentors are for different stakeholders is essential. It is also important to consider the policy and regulatory standards and expectations for a particular context as well as global best practices relevant to the revised programme.

Since the roles of many stakeholders often need to change when programmes scale up, training needs are not limited to only CHWs. In fact, some of the most important training related to scaling CHW programmes is often training for other cadres of PHC workers, such as nurses and physicians, and supervisors and managers of health systems from the district up to national level. Training these other cadres can help them understand how to effectively collaborate with and support CHWs and also to avoid or allay concerns about CHWs contributing competition, confusion, or poor quality of care to the existing health system. National-level and mid-level buy-in and support for scale-up of CHWs includes training and support for the managers and supervisors that are overseeing these programmes at several levels. The entire PHC system needs to adapt as CHW programmes are introduced and scaled up.

Defining orientation and/or training requirements of each stakeholder and then determining who will be responsible for designing and delivering the orientation and training is the second critical consideration. Attention to who is best-placed and appropriate to teach CHWs is important in ensuring context-specific content [125]. Some of the key trainers that have been found to be appropriate include senior CHWs, experts with first-hand experience of the context where these workers are based, community leaders, and others with specific technical or other skill sets [126–128]. Training for other clinical cadres and managers can include sharing and learning peers, experiences from working directly with CHWs, and guidance and encouragement from higher levels of government or experts from different development partners. The WHO guideline advises that faculty for the training of CHWs should ideally include other health workers, thereby facilitating the incorporation of CHWs as members of multidisciplinary PHC teams, and the faculty should also include supervisors of CHWs. The creation of a safe and supportive training environment is critical, with special attention to the needs of women trainees and of trainees who are members of minority and other vulnerable groups. Approaches for training diverse stakeholders may also include implications for setting up training-of-trainer (TOT) structures in order to scale training rapidly.

Next, scaling up of CHW programmes also needs to consider existing regulatory frameworks and changes in these frameworks that are needed. These frameworks and the policy approval processes associated with creating them have important implications for training. In addition, best practices from the WHO guideline on CHWs also need to be consulted and aligned with the national context.

The WHO guideline considers that competency-based formal certification has many benefits, including increasing the self-esteem of CHWs and the respect they receive from other health workers. It is also useful as CHWs transfer to other sites and other employers, and as they apply for further education and training. It provides protection to the public from those who do not have the requisite skills and training yet purport to be qualified. And it can provide a basis for reimbursement for CHW services. Finally, WHO recommends that efforts be made to establish a formal accreditation process for educational institutions that provide training for CHWs [37]. In addition to global and national policies to guide training at scale, considering roles for village health councils or village health committees in the training process can be valuable to provide local context and community-level engagement [129, 130]. These committees also often include CHWs as members.

We recognize several important limitations of our paper. While the paper covers a lot of ground, it cannot explore every aspect of CHW training in depth, and for some countries, even basic data were missing. Further, the case examples of training in Ethiopia and Bangladesh and for the ongoing COVID-19 pandemic provide a more in-depth view of training of CHWs, but many more case examples could be cited. Finally, we acknowledge that this paper was not able to make specific recommendations for CHW training programmes given the wide variation in programme contexts, approaches, and goals.

Finally, in order for CHW programmes to sustainably scale up high-quality training, adequate resources are necessary. Building the scaled-up training programme and the evidence of impact is often an iterative process that includes experimenting with different approaches and evaluating what is working well and where challenges arise in order to modify accordingly [131].

The rapid progression of the COVID-19 pandemic throughout the world and the need of CHWs to quickly acquire the skills they need to contribute to pandemic control led to challenges and opportunities that are relevant to this paper. Box 3 provides an overview of some of the training approaches used at scale in CHW programmes during the COVID-19 pandemic.

**Table:** 

**Key Message 3**
Scaling up CHW training requires providing knowledge and skills not only to CHWs, but also to ensuring that other cadres that they work with and relevant regulatory bodies are prepared to acknowledge, certify, and integrate these workers.

Box 3: Training CHWs for their roles in the COVID-19 response Many countries have been relying on existing PHC systems and CHW programmes in order to roll out necessary COVID-19 response efforts. From contexts across all regions of the world and including low-, middle-, and high-income contexts, engaging existing CHWs and also sometimes adding additional workers in CHW-type roles have enabled COVID-19 functions to be implemented rapidly and at the community level. This is an example of scaling up existing structures and models by adding new roles for current workers and also reaching new populations with specific services.

Iran’s CHWS (*behvarzs* and *moraghebe-salamats)* were trained to identify potential cases and carry out contact tracing [132]. In early 2020, they screened 76 million people (92% of the total population) over a 2-month period, referred suspected cases, and trained others in contact tracing. This reduced the workload at hospitals. *Behvarzs* and *moraghebe-salamats* also helped to promote social distancing during the pandemic. In Brazil, one of the countries hit hardest by COVID-19, CHW unions have also exposed some of the challenges and limitations of response efforts—such as lack of training and lack of national guidelines for providing PHC services [133]. They have been vocal about the impacts of this lack of guidance and support in terms of threats, deaths, and suboptimal ability to provide appropriate and necessary services to the populations they serve [133]. Liberia’s almost 4000 CHAs all received a standardized virtual 5-day course on how to respond to the COVID-19 pandemic (R. Panjabi, personal communication, 2020).

Individualized online training using Android cell phones has now been developed for CHWs to obtain training on the COVID-19 response and has been given to 8000 CHWs in Sierra Leone, building on previous experience in training CHWs to respond to the Ebola epidemic [134]. These mobile trainings bring many advantages to the traditional in-person trainings. CHWs can listen to them at convenient times, a great advantage for women particularly. The trainings can be readily translated to the local language. Persons living in isolated areas can readily access them. The training costs are reduced substantially. Pre-tests and post-tests can be incorporated into the trainings, the trainings can be adjusted if need be, and CHWs can listen to the training more than once if desired (R. Kanwagi, personal communication, 2020).

Several countries, including Liberia and South Africa, had prior experience with CHWs doing contact tracing for other disease outbreaks such as Ebola and TB [135, 136]. Since CHWs were already in place in these contexts [137], and since both the workers and the health systems had experience implementing these essential services, getting workers mobilized, maintaining community trust, and being able to provide clear and simple messages and information rapidly has greatly supported the early COVID-19 response in these contexts [135, 136].

In high-income contexts, including the United Kingdom and the United States, CHWs are being recognized as key personnel to implement strategies needed to control and suppress cases of COVID-19 [12, 138]. In addition, projects in these contexts are also recognizing that COVID-19 is an opportunity to continue to scale up and emphasize the value of CHWs in the longer term [138, 139].

In all cases, there is a need to provide training quickly at scale to existing CHWs as well as to new CHWs recruited specifically to respond to the COVID-19 pandemic. In these cases, the development of videos that can be shared with CHWs through the Internet using cell phones and/or tablets makes it possible for standardized training to be disseminated quickly. More opportunities are rapidly being developed for this, including Amref Health Africa’s Leap application [140] and the COVID-19 Digital Classroom [141]. These experiences will be useful in improving in-service training and continuing education to large numbers of CHWs in national programmes.

## Conclusion

This paper has laid out a set of considerations across three broad themes—professionalization, quality and performance, and scale-up—related to training for CHWs. While every context is different and requires consideration for how approaches need to be adapted, as evidence continues to mount some cross-cutting approaches and considerations are becoming clear. Currently, there is a great deal of support and enthusiasm for CHW programmes. If this can be leveraged now to further embed well-trained CHWs into strong PHC systems, their contributions and impact will support continued future investment and action to ensure that they remain a critical and well-supported cadre of the PHC workforce.

Training is a comprehensive and dynamic element of CHW programmes that needs to be well funded and professionalized (meaning that there is ongoing assessment of quality and continuous quality improvement). Training must be seen as much more than just pre-service training, but rather as ongoing iterative training. Training for effective CHW programmes also needs to be seen not only as training of CHWs but also as training the community, supervisors of CHWs, and others within the health system in order to help these stakeholders appreciate, understand, and make effective use of CHWs.

CHW roles will continue to change over time, and therefore ongoing and dynamic training updates will be an essential element of an effective CHW programme. This includes adding trainings for new evidence-based interventions and approaches in response to unmet, new, and emerging population health needs. Public health emergencies, such as the COVID-19 pandemic, bring urgent further attention to the need for ongoing and responsive training of CHWs.

## Data Availability

All of the data and findings reported in this paper are from appropriately cited sources for from the personal experience of the authors and their colleagues.

## Abbreviations

ANM: Auxiliary nurse midwife
ASHA: Accredited social health activist
CHV: Community health volunteer
CHW: Community health worker
COVID: Coronavirus disease
HBNC: Home-based neonatal care
HEW: Health extension worker
HIV: Human immunodeficiency virus
iCCM: Integrated community case management (of childhood illness)
MNCH: Maternal, neonatal, and child health
NCDs: Noncommunicable diseases
NGO: Nongovernmental organization
PHC: Primary healthcare
SDGs: Sustainable Development Goals
TB: Tuberculosis
TOT: Training of trainers
WDA: Women’s Development Army
